# An Antenna Array with Wide Flat-Top Beam and Low Sidelobes for Aerial Target Detection

**DOI:** 10.3390/s25195991

**Published:** 2025-09-28

**Authors:** Liangzhou Li, Yan Dong, Xiao Cai, Jingqian Tian

**Affiliations:** 1 Research Center of Applied Electromagnetics, Nanjing University of Information Science and Technology, Nanjing 210044, China; llz202212490159@126.com (L.L.); dongyan6713@126.com (Y.D.); caixiao@nuist.edu.cn (X.C.); 2 State Key Laboratory of Millimeter Waves, Southeast University, Nanjing 210096, China; 3 Department of FinTech, Nanjing University of Information Science and Technology, Nanjing 210044, China

**Keywords:** beampattern synthesis, flat-top pattern, microstrip array, sidelobe level, target detection

## Abstract

The misuse of drone technology poses significant risks to public and personal safety, emphasizing the need for accurate and efficient aerial target detection to prevent detection failures due to randomly distributed airborne targets and mitigate interference from undesired directions. Unlike conventional beam-synthesis techniques that often require either a large number of array elements or iterative numerical optimization, the proposed method analytically derives the excitation distribution by solving a newly formulated weighted-constraint problem, thereby fully accounting for mutual coupling between elements and ensuring both computational efficiency and design accuracy. In this communication, a 10 × 4 planar microstrip antenna array with a wide flat-top beam and low sidelobe is designed based on the extended method of maximum power transmission efficiency. The optimized distribution of excitations for the antenna array, which achieves a shaped beam with uniform gain over the desired angular range while suppressing sidelobe levels (SLLs) outside the shaped region, is derived by analytically solving a newly formulated weighted constraint problem. To reduce the number of antenna elements and enhance radiation characteristics, the inter-element spacings in the E-plane and H-plane are set to 0.55 λ_0_ and 0.75 λ_0_, where λ_0_ is the free-space wavelength at 3.5 GHz. Measurement results indicate that the flat-top beam in the E-plane has a wide half-power beamwidth (HPBW) of 51.2° and a low SLL of −30.1 dB, while the beam in the H-plane has a narrow HPBW of 20.1° and a low SLL of −30.5 dB, thereby demonstrating its capability in aerial target detection and airborne tracking applications.

## 1. Introduction

Sector beam antenna arrays can generate a wide beam in one plane while producing a narrow beam in the orthogonal plane [1], which have been widely applied in imaging [2,3], radar systems [4], obstacle detection [5], and wireless communications [6,7,8,9], including millimeter-wave multiple-input–multiple-output (MIMO) radar for autonomous driving and high-capacity 5G/6G links [8,9]. However, the conventional sector beam antenna arrays used in multiple target communications lack precise beamforming and -effective sidelobe suppression techniques, leading to inefficient and inaccurate track or detection due to the randomly distributed targets in a wide spatial range and the interference from undesired directions.

The common way to achieve a sector beam is to use lens antenna [10], substrate-integrated waveguide (SIW) slot antennas [6], and microstrip antennas [11,12,13]. In [14], a sector beam with high gain is realized based on SIW, offering seamlessly integration with other circuit components. A single-layer, dual-band, shared-aperture array with a small frequency ratio is reported in [15], demonstrating excellent performances of broad coverage, high resolution, and high gain. Most of the studies on sector beam focus on obtaining a pencil beam with wide half-power beamwidth (HPBW) or high gain, without considering beam shaping. For uniform radiation coverage over the target region, an antenna array with a wide flat-top beam with uniform gain is preferable. A 6-element microstrip antenna array with wide flat-top angular coverage is proposed in [12]. Similarly, a Fabry–Pérot cavity antenna with broad flat-top coverage in both the E-plane and H-plane is presented in [16]. While these designs offer wide detection coverage, the slow attenuation in the transition region between the main lobe and sidelobe, along with high sidelobe levels (SLLs), may reduce the resolution capacity of the main beam and cause inevitable interference [17,18], as highlighted in recent studies on high-resolution automotive radar and spectrum-sharing systems. To enhance detection accuracy, a slotted ridge waveguide antenna array with a flat-top beam and low sidelobes based on a pattern synthesis method is proposed in [19], achieving a HPBW of 54.4° and a fast attenuation of 17.8° in the E-plane. The design shows the SLL of −19.6 dB, which is relatively high, and a nonuniform gain distribution. In addition, a quadratically constrained quadratic programing (QCQP) based optimization approach for synthesizing flat-top radiation patterns in both far-field and near-field regions has been demonstrated, where the problem is linearized for efficient implementation and experimentally validated by base station and RFID reader antennas with less than 1–3 dB gain fluctuation [20]. In [21], an optimization algorithm is introduced to minimize the maximum SLL of an unmanned aerial vehicle array under main lobe ripple constraints. Other classic algorithms, such as genetic algorithm [22], particle swarm optimization [23], and simulated annealing [24] are also effective for obtaining optimal distribution of excitations (ODE) for specific radiation pattern specifications. Despite their flexibility in addressing various practical requirements, these algorithms require long iteration time and substantial computational resources, especially as the number of array elements increases.

In this communication, a 10 × 4 planar microstrip patch array with wide sector beam and low sidelobes is designed based on the extended method of maximum power transmission efficiency (EMMPTE). In order to achieve a wide flat-top beam in the desired angular coverage while suppressing all SLLs outside the shaped region, a new weighted constraint problem is proposed and solved analytically, yielding the ODE for the antenna array. The inter-element spacings in the E-plane and H-plane are optimized to obtain a better radiation characteristic while reducing the number of antenna elements. Measurement results indicate that a wide flat-top beam with an HPBW of 51.2° and a gain fluctuation less than 1 dB in the E-plane is achieved. The measured SLLs of the E-plane and H-plane maintain below −30.1 dB and −30.5 dB, respectively.

## 2. Design Method

**Figure 1:**
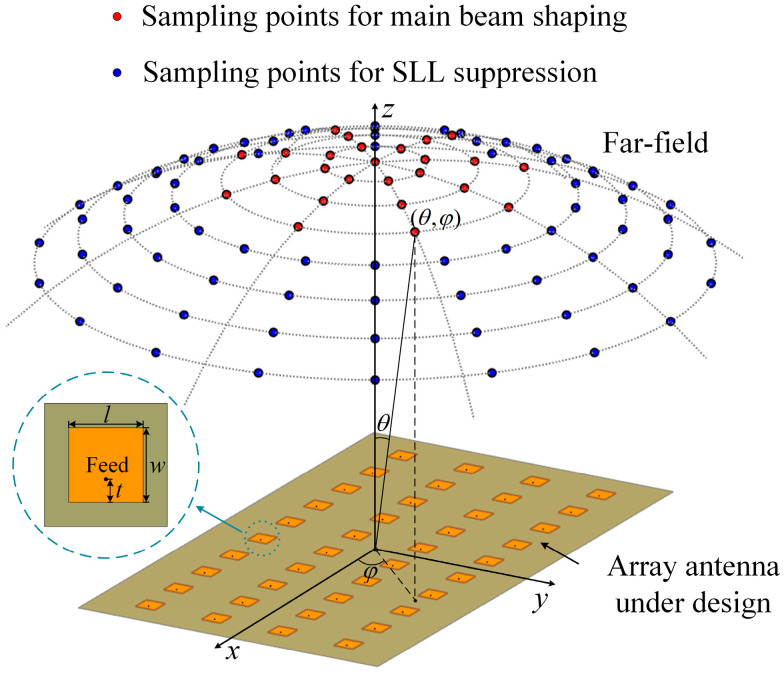
Diagram of the proposed method.

Consider an unevenly spaced planar array composed of n elements, as shown in Figure 1. It is assumed that the region to be shaped is located in the far field of the array and is uniformly discretized into a number of grid points.

In [25], various focused near-field patterns are produced by introducing different weighted constraints based on EMMPTE. However, the method proposed in [25] focuses on shaping the main lobe pattern by maximizing energy concentration, without imposing any explicit constraints on sidelobe suppression. In practical applications, the reduction in SLLs is important, as it effectively minimizes interference and noise in directions outside the main lobe region, thereby enhancing the overall performance of the system. This motivates our work.

**Figure 2:**
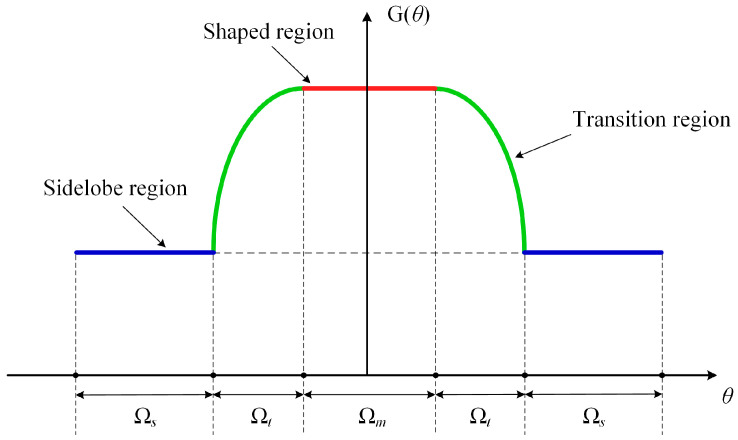
Schematic of the division of regions in the radiation pattern.

This communication proposes a new constrained optimization method based on EMMPTE, in which the main lobe beamforming is enforced through a constraint to generate the desired shape, while the SLLs are effectively suppressed via the objective function to meet practical application requirements. To precisely control its shape, the radiation pattern is divided into several blocks for independent adjustment. As shown in Figure 2, the radiation pattern can be divided into three regions: the shaped region Ω*_m_*, the transition region Ω*_t_* and the sidelobe region Ω*_s_*. To ensure that the main lobe beamforming is not affected while reducing the SLLs, the objective function is set to minimize the energy in the sidelobe region. Subsequently, a constraint involving an m-dimensional weighting vector is introduced to regulate the energy distribution within the main lobe shaping region. Additionally, since the formulation is normalized, any constant scaling of [*w*] yields the same normalized solution, and thus the absolute value of the constant does not affect the beam flatness. Incorporating this constraint with the objective of minimizing energy in the sidelobe region, the following constrained optimization problem is formulated:

(1) $$\begin{array}{l} \min\;[a_{t}{]}^{H}[B][a_{t}]\\ s.t.\;[e_{t}][a_{t}]=[w], \end{array}$$

where $[a_{t}]={\left[a_{1},a_{2},\ldots ,a_{n}\right]}^{T}$ is the complex excitation vector; $[e_{t}]=\left[\left[e(\theta_{1},\varphi_{1})],[e(\theta_{2},\varphi_{2})],\ldots ,[e(\theta_{m},\varphi_{m})\right]\right]$ is *m* × *n* matrix of electric field distribution along *m* sampling points in the shaped region; $\left[e(\theta_{i},\varphi_{i})]=[e_{1}(\theta_{i},\varphi_{i}),e_{2}(\theta_{i},\varphi_{i}),\ldots ,e_{n}(\theta_{i},\varphi_{i})\right]$ is 1 × *n* matrix of the element radiation fields, with $e_{j}(\theta_{i},\varphi_{i})$ being the electric field in the direction $\left(\theta_{i},\varphi_{i}\right)$ generated by the *j*th element excited by $a_{j}=1$ while all the other elements are terminated in matched loads; $\left[B]={\left[{\tilde{e}}_{t}\right]}^{H}[{\tilde{e}}_{t}\right]$ is the total energy in the sidelobe region and $\left[{\tilde{e}}_{t}\right]$ is a matrix of the electric field distributions along *s* sampling points in the sidelobe region; the superscripts “*T*” and “*H*” represent the transpose and conjugate transpose operation. The above problem can be solved analytically by using the Lagrange multiplier method [25,26,27], and the optimal solution to Equation (1) is given by:

(2) $$[a_{t}]={\left[B\right]}^{-1}{\left[e_{t}\right]}^{H}{\left([e_{t}]{\left[B\right]}^{-1}{\left[e_{t}\right]}^{H}\right)}^{-1}[w].$$

By adjusting the weighting coefficients in [w], various shaped patterns can be achieved. For example, the flat-top beam can be realized by setting the weighting vector [w] to be a constant vector. To further control the beam shape precisely, such as generating a secant-squared beam, the weighting coefficients can be selected according to the desired field pattern.

Additionally, mutual coupling is inherently accounted for by extracting the coupled fields of all elements from full-wave simulations and analytically solving the weighted-constraint problem, ensuring a unique and physically accurate excitation distribution. Furthermore, the objective function adopted in this work is fundamentally distinct from that in [25,26,27], thereby defining different optimization problems and targeting different application scenarios such as mainlobe beam shaping, multibeam generation, and sidelobe suppression. In particular, while [25] formulates its objective function to maximize the total radiated energy within the mainlobe region, it does not include an explicit mechanism to suppress SLLs. As a result, the synthesized patterns in [25] may exhibit elevated SLLs, which can lead to degraded interference rejection and reduced detection ac-curacy in practical applications such as radar, wireless communication, and aerial target detection, where stringent SLLs control is critical. This motivates the proposed Formulation (1), which explicitly minimizes the total electric-field energy in the sidelobe region while simultaneously enforcing a prescribed energy distribution within the mainlobe region via a weighted constraint.

This dual-objective design ensures that the resulting excitation distribution achieves both flat-top mainlobe shaping and deep sidelobe suppression without requiring additional iterative optimization or post-processing steps. Consequently, the proposed method not only provides a closed-form analytical solution that is computationally efficient, but also guarantees superior performance in scenarios demanding low SLLs and high radiation pattern fidelity.

## 3. Sector Beam Antenna Array Optimization

**Figure 3:**
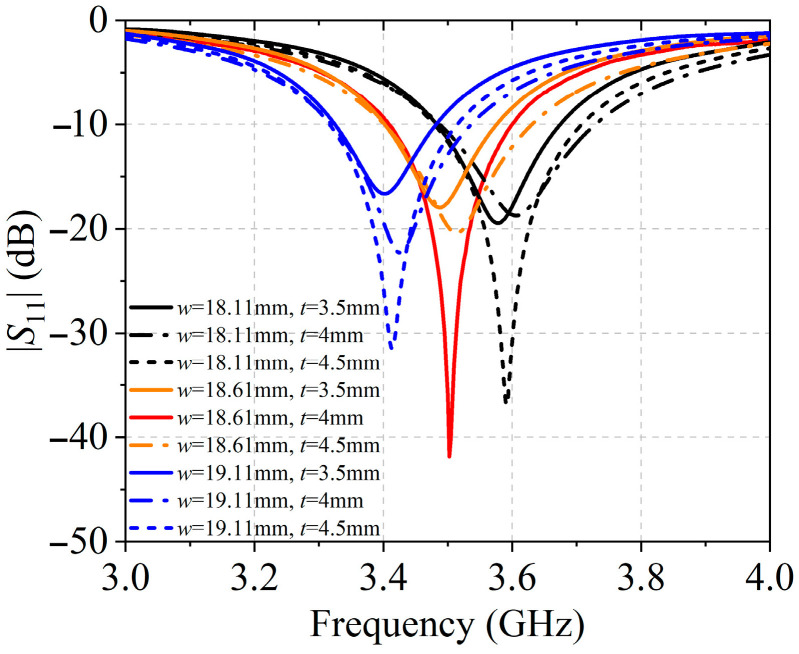
Simulated reflection coefficients for the antenna element with different *w* and *t*.

The design of the square patch antenna operating at 3.5 GHz was carried out through a parametric optimization using HFSS software (version 16.1.0). As shown in Figure 3, the patch length *w* and the feed inset position *t* were systematically varied to achieve optimal impedance matching. Specifically, three candidate values for *w* = 18.11 mm, 18.61 mm, and 19.11 mm and three feed positions *t* = 3.5 mm, 4 mm, and 4.5 mm were investigated, resulting in 9 combinations and their corresponding reflection coefficients. The combination *w* = 18.61 mm and *t* = 4 mm provided resonance precisely at 3.5 GHz with the lowest return loss, thus offering the best impedance matching and forming the basis for the final antenna element dimensions. The optimization of inter-element spacing was jointly guided by the practical structural constraints of the array and the beam shaping requirements dictated by the target application. The proposed antenna array adopts a 4 × 10 configuration, with four elements along the *y*-axis and ten elements along the *x*-axis, and the spacings in both directions were systematically optimized, as shown in Figure 1.

**Figure 4:**
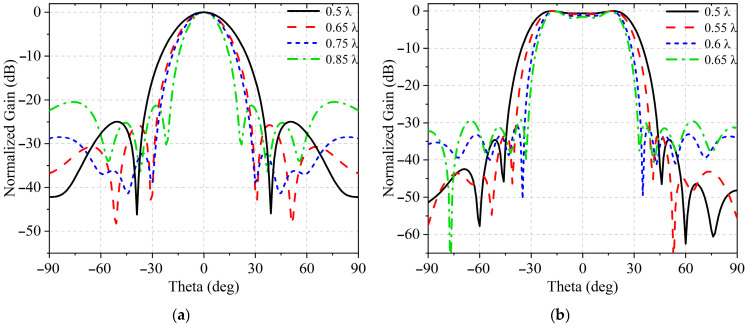
Simulated normalized radiation patterns in (**a**) H-plane; (**b**) E-plane.

For the *y*-axis, where a narrower beamwidth was required, the number of elements was fixed at four due to mechanical limitations, and various spacings—0.5 λ_0_, 0.65 λ_0_, 0.75 λ_0_, and 0.85 λ_0_—were examined under identical conditions. As shown in Figure 4a and Table 1, after applying the proposed sidelobe suppression method, the resulting beam patterns were compared in terms of beamwidth, grating lobe suppression, and sidelobe performance. The configuration with 0.75 λ_0_ spacing offered the most balanced trade-off, providing effective beamwidth reduction while maintaining sidelobe control and avoiding the onset of grating lobes.

For the *x*-axis, the design objective was to realize a flat-top beam with minimal ripple and a sharp roll-off at the beam edges. Based on the selected *y*-axis spacing of 0.75 λ_0_, inter-element spacings of 0.5 λ_0_, 0.55 λ_0_, 0.6 λ_0_, and 0.65 λ_0_ along the *x*-axis were evaluated. As shown in Figure 4b and Table 2, the proposed beam-shaping and sidelobe suppression techniques were applied to each configuration, and the resulting radiation patterns were analyzed in terms of ripple amplitude within the shaped region, sidelobe level, and edge steepness. Among the evaluated options, an inter-element spacing of 0.55 λ_0_ demonstrated the lowest sidelobe level, the flattest beam profile across the desired coverage region, and the most favorable edge transition characteristics. Consequently, this spacing was selected as the optimal choice for the final array design. Regarding the proposed non-uniform array with 0.75 λ_0_ spacing in the H-plane and 0.55 λ_0_ spacing in the E-plane, it was selected because it offers the best overall balance among beamwidth, sidelobe level, ripple control, and roll-off steepness, outperforming the uniformly spaced arrays in most key performance metrics. Once the configuration of the array is determined, the electric field distribution matrix $\left[e_{t}\right]$ can be obtained through a full-wave simulation.

**Figure 5:**
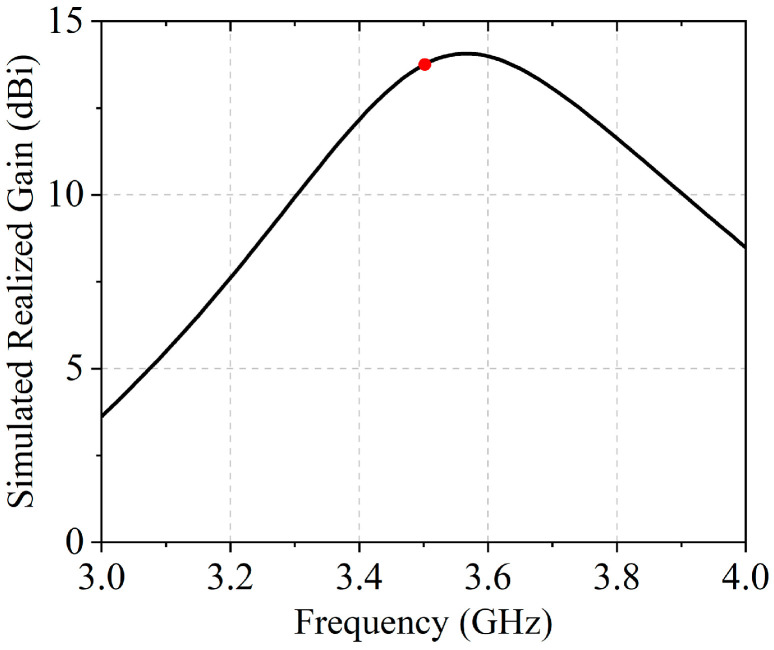
Simulated realized gain versus frequency across the operating band.

The operating band of the proposed array spans from 3.4 to 3.6 GHz, with a center frequency at 3.5 GHz. As indicated in Figure 5, the simulated realized gain reaches its maximum of 14 dBi at 3.55 GHz, while a gain of 13.4 dBi is achieved at 3.5 GHz and highlighted by the red dot.

**Figure 6:**
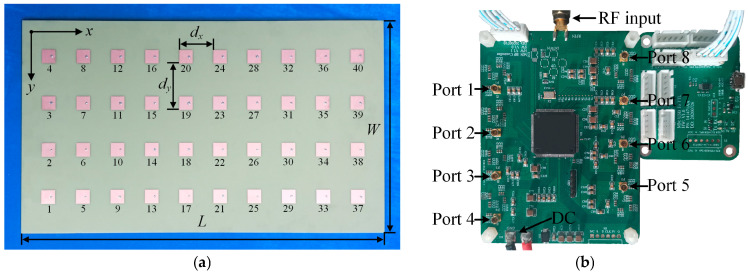
The fabricated (**a**) 10 × 4 antenna array and its corresponding; (**b**) adjustable 8-channel RF feeding circuit.

As shown in Figure 6a, the proposed array, with overall dimensions of *L* = 497.06 mm and *W* = 291.38 mm, element spacings of *d_y_* = 64.275 mm and *d_x_* = 47.135 mm, and fabricated on a *h* = 3 mm FR4 substrate (*ε_r_* = 4.35, and a loss tangent of 0.025), is prototyped and tested to verify the beamforming and SLLs suppression performance. A digital RF circuit is shown in Figure 6b and is designed to feed the antenna array with the ODE.

The feeding circuit consists of four 8-channel digital beamforming controllers interconnected via bidirectional power splitters. Each channel’s attenuation and phase shift can be independently controlled using a 6–bit attenuator with a maximum attenuation value of 31.5 dBi and a 6–bit phase shifter with a phase resolution of 5.625° integrated with the transceiver chip. Control signals are transmitted through the serial peripheral interface module, allowing each channel’s excitation to be individually programmed. During the test, the RF feeding circuit is connected to the array via coaxial cables, and the unused channels are terminated with matched loads. For each port, the deviation between ODE and the actual excitation implemented by the RF feeding circuit is strictly limited to be less than 0.01 V in amplitude and 4° in phase, ensuring accurate beam shaping performance.

## 4. Results and Discussion

### 4.1. Simulation and Measurement Results

**Figure 7:**
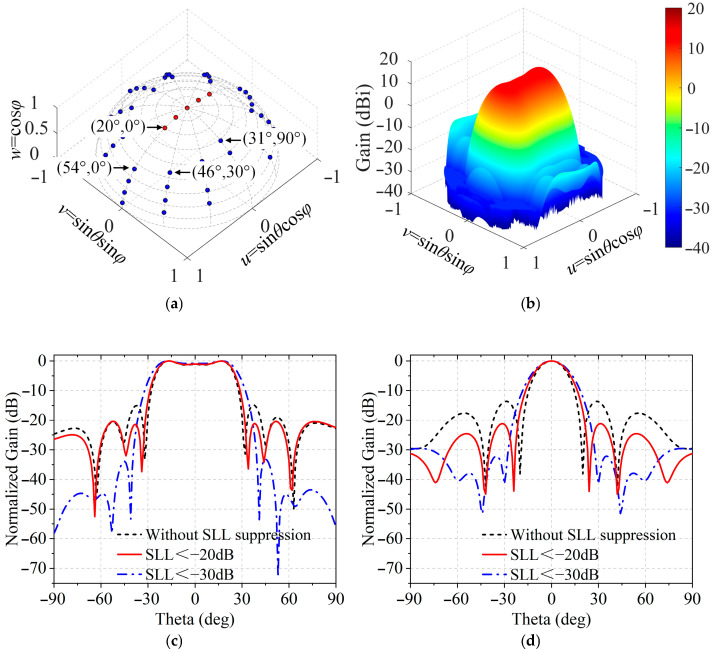
Simulated beam shaping radiation patterns at 3.5 GHz with (**a**) distribution of sampling points, (**b**) 3D radiation pattern and 2D radiation patterns in the (**c**) E-plane and (**d**) H-plane.

Figure 7a illustrates the specific sampling points for synthesizing the flat-top pattern with suppressed SLLs. The sidelobe regions are defined as [−90°,−54°]∪[54°,90°] and [−90°,−31°]∪[31°,90°] in E-plane and H-plane, respectively. The sidelobe sampling points are distributed with a spacing of 1°. The ODE listed in the third column of Table 3 is calculated by (2) and the 3D gain pattern is plotted in Figure 7b. Figure 7c,d reveal that when SLL < −30 dB, the array exhibits a gain of 13.4 dBi, a HPBW of 52.2° and the SLL of −33.1 dB in E-plane, and achieves a slightly lower gain of 12.6 dBi, a narrower HPBW of 20.9°, and the SLL of −32.1 dB in H-plane. Compared to the results obtained by the previous method in [25] without sidelobe suppression, the SLLs of the proposed design are remarkably reduced by 17.7 dB and 18.5 dB. Additionally, a 5.1° wider HPBW in the E-plane is achieved, with only a slight reduction of 1.3 dB in gain and when SLL < −20 dB, the drop of gain is 0.4 dB.

**Figure 8:**
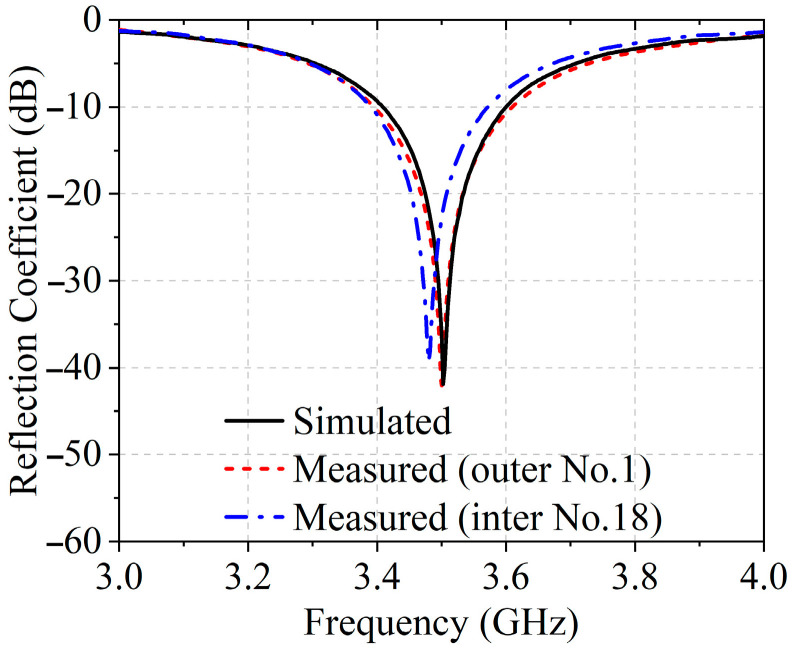
Simulated and measured reflection coefficients for the antenna element.

**Figure 9:**
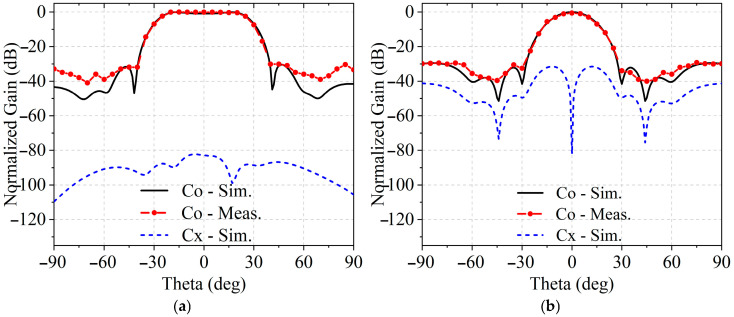
Measured and simulated normalized radiation patterns at 3.5 GHz in (**a**) E-plane and (**b**) H-plane.

The measured reflection coefficient of the patch element, shown in Figure 8, indicates good impedance matching at 3.5 GHz. The reflection coefficient was measured for element No. 1 and, for comparison, an inner element No. 18 with relatively stronger coupling. The inner element exhibits a slight leftward shift of 20 MHz due to mutual coupling, yet the measured reflection coefficient at 3.5 GHz remains −20.2 dB, indicating negligible impact. Element No. 1 shows close agreement with simulation, reflecting minimal inter-element interaction. These results confirm that the array preserves accurate active performance across its elements. The overall simulated radiation efficiency of the array to be approximately 65.1%. The measured radiation patterns, shown in Figure 9a,b, reveal that the HPBW in the E-plane and H-plane are 51.2° and 20.1°, respectively. The corresponding SLLs are measured to be −30.1 dB and −30.5 dB. As can be seen from Figure 9, the cross-polarization levels are very low, indicating excellent polarization purity of the array. The measured and simulated results show good agreement, with minor deviations in SLLs attributed to the limited phase accuracy of the RF circuits caused by the inherent precision constraints of the phase shifters. Due to the limitations of the experimental environment and measurement conditions, signals below approximately −40 dB after normalization are nearly indistinguishable from environmental noise, and the flat-top beam in the E-plane naturally generates more low-level sidelobes below −40 dB than in the H-plane, resulting in slightly less accurate measurements for the E-plane. Both the measurement and simulation results confirm that the proposed method not only synthesizes a wide flat-top pattern but also effectively suppresses SLLs as expected.

### 4.2. Comparison and Discussion

Table 4 provides a comparison between published designs and the proposed design from several aspects, including array dimensions, beamwidth, attenuating capacity, gain fluctuation and SLL. Here, the gain fluctuation is defined as the maximum deviation of the gain inside the shaping region, and is used as an indicator to evaluate the effectiveness of flat-top beam shaping approaches.

The proposed design has at least three notable advantages based on the comparison. First, it achieves the best flat-top shaping effect with the gain fluctuation less than 0.4 dB in the beam shaping region. Second, the proposed design demonstrates excellent attenuating capacity. Here, the attenuating capacity is quantified by the angular interval corresponding to the drop of the radiation pattern from −3 to −15 dB. Unlike the classical first-null beamwidth, this metric is more indicative of the region most susceptible to strong interference (i.e., above −15 dB), especially in scenarios such as aerial target detection, radar sensing and base station communication systems. The measured gain drops from −3 to −15 dB within only 8.63°, resulting in minimal interference outside the flat-top region compared to the other designs. Third, the SLL of −30.1 dB is the lowest among the designs. Many other designs exhibit relatively high SLLs for achieving both good beam-shaping performance and low SLLs simultaneously is challenging. Specifically, to achieve a flat-top beam, the amplitude distribution of excitations for the antenna array should meet certain requirements. However, the SLL suppression also imposes constraints on the amplitude distribution. Thus, a conflict may arise in determining the amplitude distribution for the array. The proposed design has solved this problem by introducing a new optimization problem.

## 5. Conclusions

Based on the EMMPTE, this communication introduces a novel optimization method with equality constraint to achieve a shaped sector beam with low SLLs. By minimizing the energy in the sidelobe region and regulating the energy distribution within the shaped region through specific constraints, the ODE for the antenna array under design is analytically derived. As a demonstration, a 10 × 4 microstrip antenna array is designed and tested. The measured results indicate that the 45° flat-top beam exhibits a gain fluctuation of only 0.4 dB, with the SLL reaching −30.1 dB in the E-plane and −30.5 dB in the H-plane, making it highly suitable for aerial target detection and airborne tracking applications.

## Figures and Tables

**Table 1:** Performance comparison among different inter-element spacings along *y*-axis.

Spacing (λ_0_)	HPBW (°)	SLL (dB)
0.5	28.7	−25
0.65	22.7	−25.7
0.75	21	−33.3
0.85	17.2	−21.3

**Table 2:** Performance comparison among different inter-element spacings along *x*-axis.

Spacing (λ_0_)	Beamwidth (°)	SLL (dB)	Attenuating Capacity (°)	Fluctuation (dB)
0.5	56.3	−36.5	10	0.79
0.55	52.2	−33.1	8.97	0.76
0.6	47.4	−29.8	6.8	1.34
0.65	44.9	−29	6	1.97

* Attenuating capacity: the degree corresponding to the drop of the radiation pattern from −3 dB to −15 dB.

**Table 3:** Distribution of excitations.

Port No.	ODE Without SLL Suppression	ODE with SLL < −30 dB
1, 4	0.049 ∠ −157°	0.009 ∠ −171.4°
2, 3	0.050 ∠ −159.3°	0.034 ∠ −170.4°
5, 8	0.075 ∠ 15.7°	0.008 ∠ −2.6°
6, 7	0.076 ∠ 13.9°	0.023 ∠ −0.7°
9, 12	0.069 ∠ 4.4°	0.038 ∠ 12.3°
10, 11	0.070 ∠ 4.9°	0.109 ∠ 10.6°
13, 16	0.121 ∠ −178.4°	0.041 ∠ 180.0°
14, 15	0.123 ∠ −179.4°	0.086 ∠ 178°
17, 20	0.312 ∠ −173.1°	0.179 ∠ −174.0°
18, 19	0.316 ∠ −174.7°	0.444 ∠ −174.5°
21, 24	0.307 ∠ −173°	0.177 ∠ −174.3°
22, 23	0.313 ∠ −174.8°	0.439 ∠ −174.2°
25, 28	0.115 ∠ −177.3°	0.037 ∠ −176.9°
26, 27	0.119 ∠ 178.8°	0.078 ∠ 178.6°
29, 32	0.073 ∠ 3.5°	0.038 ∠ 12.6°
30, 31	0.072 ∠ 4.8°	0.110 ∠ 10.7°
33, 36	0.072 ∠ 18.4°	0.006 ∠ 1.9°
34, 35	0.074 ∠ 15.3°	0.020 ∠ −0.7°
37, 40	0.052 ∠ −157.3°	0.010 ∠ −171.3°
38, 39	0.053 ∠ −158.5°	0.036 ∠ −170.4°

**Table 4:** Performance comparison among different antenna arrays.

Ref.	Type	($\mathrm{Dimensions}\;\lambda_{0}^{3}$)	Beamwidth (°)	Attenuating Capacity (°)	Fluctuation (dB)	SLL (dB)
[28]	Patch	1.93 × 1.93 × 0.075	80_3dB_	16	1.51	−18.3
[16]	Patch	2.37 × 2.37 × 0.67	58_3dB_	19.5	1.6	−13.2
[29]	Patch	4.14 × 4.14 × 0.31	54.9_3dB_	10.2	1.97	−20
[30]	Leaky Wave	4.45 × 4.45 × 0.43	40_3dB_/34_1dB_	18	1	−15.4
[10]	Lens	15 × 10 × 0.9	50.2_3dB_/30.4_1dB_	28.9	—	−15
[19]	SIW	26.4 × 5.3 × 0.24	54.4_3dB_	13.2	1.3	−19.3
[31]	SIW	3.82 × 1.94 × 0.14	50_3dB_	15.7	1.16	−16.7
[32]	Dipole	3.19 × 3.19 × 1.3	45_3dB_	13.9	1.38	−21.1
This work	Patch	5.8 × 3.2 × 0.035	48.2_3dB_	6.5	1.2	−20.4
			51.2_3dB_/45_1dB_	8.63	0.4	−30.1

* Attenuating capacity: the degree corresponding to the drop of the radiation pattern from −3 dB to −15 dB.

## Data Availability

Data are contained within the article.
